# Enhancing Physical Fitness in Primary School Children Through Inclusive Sports Activities

**DOI:** 10.3390/children12060758

**Published:** 2025-06-11

**Authors:** Nikola Utvić, Lidija Marković, Radenko Arsenijević, Nikola Aksović, Bojan Bjelica, Stanimir Stojljković, Saša Bubanj, Gabriela Raveica, Daniel-Lucian Dobreci, Nicolae-Lucian Voinea, Vasile-Cătălin Ciocan, Mihaela Anghel, Bogdan-Alexandru Antohe, Tatiana Dobrescu

**Affiliations:** 1 Faculty of Sport and Physical Education, University of Priština-Kosovska Mitrovica, 38218 Leposavić, Serbia; nikola.utvic@pr.ac.rs (N.U.); radenko.arsenijevic@pr.ac.rs (R.A.); kokir87np@gmail.com (N.A.); 2 Faculty of Sport and Physical Education, University of Novi Sad, 21000 Novi Sad, Serbia; markoviclidija169@gmail.com; 3 Faculty of Physical Education and Sport, University of East Sarajevo, 71420 Pale, Bosnia and Herzegovina; vipbjelica@gmail.com; 4 Faculty of Sport and Physical Education, University of Belgrade, 11000 Belgrade, Serbia; stanimirstojiljkovic@gmail.com; 5 Faculty of Sport and Physical Education, University of Niš, 18000 Niš, Serbia; 6 Faculty of Movement, Sports, and Health Sciences, “Vasile Alecsandri” University of Bacău, 600115 Bacău, Romania; gabriela.raveica@ub.ro (G.R.); dobreci.lucian@ub.ro (D.-L.D.); ciocan.catalin@ub.ro (V.-C.C.); anghel.mihaela@ub.ro (M.A.); antohe.bogdan@ub.ro (B.-A.A.); tatiana.dobrescu@ub.ro (T.D.)

**Keywords:** motor abilities, Eurofit test battery, physical education, children, adaptive sports, inclusive activities

## Abstract

(1) Background: The aim of this study is to investigate whether the application of adaptive sports programs, initially designed for children with disabilities, can have a positive effect on physical fitness and body composition in healthy primary school children; (2) Methods: The sample comprised 80 participants, equally divided by gender (50% male, 50% female), with a mean age of 11.5 (SD = 0.03). They were divided into two groups, an experimental and a control group, with an even gender distribution. The research lasted 12 weeks, with additional classes allocated for the initial and the final measurements. The Eurofit test battery assessed physical fitness; (3) Results: ANCOVA revealed significant group differences in body composition variables within the total sample and gender distribution (*p* < 0.001). Significant differences were observed in handgrip strength (HGR), flamingo balance test (FBL), shuttle run 10 × 5 m (SHR) (*p* < 0.001), and also hand tapping (HTP) (*p* = 0.01). Participants in the experimental group outperformed the control group, highlighting the effectiveness of the intervention program. Specifically, boys in the experimental group showed significant improvements in HGR and SHR (*p* < 0.001), while girls improved significantly in HGR, FBL, and SHR (*p* < 0.001); (4) Conclusions: The experimental program, which incorporates sports elements for disabled individuals, led to significant improvements in the physical fitness parameters of children.

## 1. Introduction

Physical fitness, a comprehensive indicator of the body’s functions during physical activities or exercise, is defined by two crucial dimensions [1]. One is performance-related, crucial for success in sports, skill-based assessments, or specific professional demands. The other is health-related, encompassing cardiorespiratory, musculoskeletal, and morphological components that contribute to an optimal health profile and are positively associated with children’s performance in physical education classes [2]. The development and maintenance of these fitness components are correlated with an individual’s level of physical activity [3]. However, it is alarming that despite inherent biological inclinations toward movement, recent decades have witnessed a noticeable decline in children’s physical activity levels, which has reduced their overall physical fitness. This decline should be a cause for concern and a call to action for all of us [4].

The literature documents numerous programs that aim to enhance physical fitness in children and adolescents, such as school-based exercise interventions, organized sports activities, and public health campaigns promoting increased levels of daily physical activity [1,5]. However, a significant gap exists in the field, as there is a lack of programs that systematically apply principles of adaptive exercise in mainstream education. It is crucial to address this gap and implement programs that personalize physical activities according to each child’s individual needs, abilities, and current health status, thereby ensuring the effectiveness of physical fitness programs.

Adaptive exercise for children is a personalized approach that involves modifying physical activities to meet individual needs and physical fitness levels. This approach is particularly beneficial for children with specific physical, mental, or sensory needs, as it ensures safety and effectiveness in physical activities by adapting them to their unique capabilities [6]. Adaptive exercise includes low-impact aerobic exercises and functional and strength-enhancing exercises, aiming to improve overall physical fitness, coordination, flexibility, and balance [7].

Despite the origin of adaptive sports in working with children with disabilities, the basic principles of this approach, such as individualization, gradual load progressions, and emphasis on functional movement, are applicable and beneficial for all children, including healthy populations [4]. However, school programs rarely tap into the potential of such methods, leaving a significant gap in optimizing physical fitness through inclusive and adaptive approaches. This underutilization should motivate educators and policymakers to make a change. Furthermore, it is crucial to implement activities that will raise awareness about developmental disorders among school-aged children in order to reduce negative consequences such as social exclusion, influence broader societal attitudes toward disability, and educate children without disabilities that their peers with disabilities have the right to participate in physical education classes and other sports activities.

The central hypothesis of this study is that the experimental group participating in the adaptive sports program will improve body composition and physical fitness parameters more than the control group, which follows the standard physical education curriculum. This study could provide valuable insights into the potential benefits of adaptive sports. Specifically, significant positive changes in strength, balance, agility, flexibility, and body composition within the experimental group are expected.

Therefore, the aim of this study is to investigate whether the application of adaptive sports programs, initially designed for children with disabilities, can have a positive effect on physical fitness and body composition in healthy primary school children. Given the global decline in physical activity levels among children, this study investigates the benefits of integrating adaptive sports into regular physical education classes to enhance health-related fitness in school settings.

## 2. Materials and Methods

### 2.1. Participants

The study involved 80 fifth-grade students (mean age = 11 years and 6 months) from primary schools in Kosovo and Metohija, Republic of Serbia. The main criteria were regular attendance of physical education classes, absence of chronic illnesses or injuries that would restrict participation in physical activities, and parental/guardian consent for participation in the study. These students were assigned to the experimental and control groups to ensure gender balance, similar age, and initial physical fitness levels. Specifically, the experimental group comprised 40 students (20 boys and 20 girls) from the “Jovan Cvijić” primary school in Zubin Potok. Furthermore, the control group consisted of 40 students (20 boys and 20 girls) from the “Vuk Karadžić” primary school in Zvečan.

### 2.2. Measuring Procedures

All participants regularly attended physical education classes according to the official educational Plan and Program approved by the Ministry of Education, Science, and Technological Development of the Republic of Serbia, which sets the standard for physical education. Inclusion criteria specified that participants were clinically healthy throughout the study period and did not have medical exemptions from physical education classes. Additionally, participants were required to have completed the initial and final measurements and maintained class attendance of at least 70%.

The experimental group was provided with a specialized program tailored to their age group, consisting of three classes per week that incorporated elements of sports adapted for individuals with disabilities. To minimize the potential influence of teacher-related effects, all physical education teachers involved in the experimental program received prior training and detailed written instructions on program implementation. A standardized curriculum was followed across all participating schools. Conversely, the control group continued their regular activities and standard physical education classes, as prescribed by the Ministry of Education, Science, and Technological Development of the Republic of Serbia, throughout the study.

The Ethics Board of the Faculty of Sport and Physical Education at the University of Niš (8/18-01-012/20-018, approval date: 25 December 2020) played a crucial role in the study, granting ethical approval for the study and ensuring adherence to the principles outlined in the Helsinki Declaration. Written consent from parents and schools was obtained before the commencement of the study, ensuring compliance with ethical standards and voluntary participation in the research.

### 2.3. Testing

The assessment involved assessing the values of individual parameters. The parameters included body weight (kg), body height (cm), Body Mass Index (BMI, kg/m^2^), percent body fat (%), and body fat mass (kg).

Body composition data were collected by measuring height and physical composition using the multi-channel bioelectric impedance method (BIA) with the InBody 270 device (Seoul, Republic of Korea). This method involves passing a low-frequency electric current through the body. The current passes through muscles without impedance (since they are well-vascularized and rich in water) but encounters resistance when passing through fat tissue (which is poor in water). This impedance is then used to calculate body composition, providing a non-invasive and quick method for assessing body fat percentage and distribution [8].

Physical fitness was comprehensively assessed using the Eurofit test battery, a thorough set of tests prescribed by the Committee for the Development of Sport [9]. The following tests were used on the initial and final measurements, ensuring a comprehensive evaluation of physical fitness: Handgrip strength—HGR (kg), Flamingo balance test—FBL (s), Hand tapping—HTP (n), Sit-and-reach—SAR (cm), Sit-ups—SUP (n), Pull-Ups Test—PUP (s), 20 m Endurance Shuttle-Run Test—BPT (ml/min/kg), Shuttle Run 10 × 5 m—SHR (s), and Standing Broad Jump—SBJ (cm).

#### 2.3.1. Handgrip Strength

This test measures the isometric strength of the upper body. The participant squeezes the grip for at least two seconds continuously, performing the test twice alternately. The optimal range is determined by the participant, a86nd a short rest is allowed between measurements. For each measurement, the first teste0d hand is chosen randomly. The elbow must be fully extended, and the dynamometer should not touch any body part except the hand being measured. Both hands are measured twice, and all results are recorded. The best attempt for each hand is taken.

#### 2.3.2. Flamingo Balance Test

This test evaluates static balance and the ability to maintain stability on one leg. The participant stands on one leg while the other leg is bent and held with the foot of the raised leg placed against the inside of the knee of the standing leg. The participant must maintain their balance as long as possible, without touching the floor with their free leg or falling. The test was performed for each leg, and the better time or performance was used as the final result.

#### 2.3.3. Hand Tapping

The hand tapping test assesses manual dexterity, reaction time, and hand-eye coordination by measuring the speed and accuracy of performing tapping or similar tasks within a set time. The student taps their dominant hand on a flat surface or a specific target as quickly as possible. The number of taps made in 15 s is counted. The number of successful taps performed in the set time is recorded.

#### 2.3.4. Sit-and-Reach

This test assesses flexibility, specifically the flexibility of the lower back and hamstrings. The participant sits barefoot in front of a box, with legs extended, placing their feet on the front side of the box. With arms extended in front (one over the other), the student leans forward as much as possible, pushing a sliding ruler forward slowly, without swinging. The result is determined by the furthest position that the student reaches in two attempts. The better result is recorded.

#### 2.3.5. Sit-Ups for 30 s

Sit-ups assess the trunk muscular endurance and strength. The participant starts lying on their back on a mat with knees bent at 90°, feet aligned with hip width, and arms crossed over their chest with palms on opposite shoulders. The examiner holds the participant’s feet to the ground. At the start signal, the participant lifts their torso into a sitting position as quickly as possible, ensuring that their elbows touch their thighs before lying back down. This lifting and returning to the starting position should be performed as fast as possible for 30 s. The result is the total number of correctly performed sit-ups.

#### 2.3.6. Pull-Ups Test

The participant holds the bar with an underhand grip at shoulder width. The chin should be above or at the height of the bar. The teacher starts the stopwatch and measures the period the student can maintain the starting position. The student must keep their legs extended and should not swing. The stopwatch is stopped when the chin drops below the height of the bar.

#### 2.3.7. Beep Test

This test evaluates aerobic capacity and cardiorespiratory endurance by requiring participants to run between two lines, 20 m apart, according to a pace dictated by an audio signal from a CD. At each audio signal, the participant must have both feet across the line. The goal of the test is running as many laps as possible. The starting speed is 8.5 km/h (20 m in 9 s), and every minute, the speed increases by 0.5 km/h. The test ends when the participant no longer can keep up with the required pace, or when they fail to cross the line three times in a row when the sound signal is heard. Each level contains a certain number of segments.

#### 2.3.8. Shuttle Run 10 × 5 m

This test implies that the participant is running as fast as possible from the starting line to the line at the other end of a 5 m track, which they must cross with both feet. Then, the participant turns around, runs back in the same direction, and stops again with both feet over the starting line (this is repeated ten times, during which they run 50 m). The time measurement starts upon the given signal and ends when the student crosses the starting line with the entire foot after completing ten 5 m sections. The sections should be run with maximum effort.

#### 2.3.9. Standing Broad Jump

The test evaluates the explosive strength of the lower extremities. The participant stands behind feet aligned with shoulder-width. In this position, the participant must bend their knees, swing their arms, push off strongly, and jump as far as possible. The participant should land on both feet and maintain an upright body position. Three attempts should be made, and the best result is recorded.

### 2.4. Experimental Program

The experimental group underwent an adaptive sports program (Table 1), while the control group continued with standard activities according to the school curriculum (elements of athletics, handball, and gymnastics).

In this study, the term “adaptive sports” refers to modified versions of conventional sports activities tailored to meet the developmental, physical, and cognitive needs of participants. These modifications, which were based on established frameworks for inclusive physical education and adaptive sports, particularly the guidelines set by the International Federation of Adapted Physical Activity, were made in terms of equipment, rules, and movement patterns to support skill acquisition and ensure safe participation. The adaptive elements were systematically incorporated into each lesson, particularly in tasks involving throwing, catching, locomotor movement, and the acquisition of sport-specific techniques.

The experimental exercise program lasted 12 weeks (36 h) and was incorporated into regular physical education classes conducted three times weekly. Each class adhered to a standard composition comprising introductory, preparatory, main, and final phases tailored to the participants’ developmental stages.

The main phase of the experimental program was purposefully devised to improve physical fitness using diverse training methods. Informed by relevant research, the intervention focused on teaching and refining sports techniques adapted for disabled individuals. It utilized instructional approaches such as direct communication and demonstration, where instructors communicated key concepts and demonstrated proper execution. Practical training sessions allowed participants to apply learned skills in a controlled setting.

The experimental sessions included tossing, crawling, and controlled falls to enhance throwing, catching, and maneuvering skills. Additionally, methodical exercises should improve jumping, shooting accuracy, and running endurance, incorporating varying forms and intensities. Throughout the program, activity complexity and intensity gradually increased to sustain participant motivation and engagement, aiming to promote independence and effective use of space and equipment during training.

The intervention was integrated into the current physical education curriculum to align with educational objectives and standards. By emphasizing enhancing physical fitness through specialized sports techniques, the program sought to optimize physical fitness outcomes for all participants.

### 2.5. Statistical Analyses

The statistical analyses involved a comprehensive process of estimating descriptive statistics, such as mean (M), standard deviation (SD), and the minimum (Min) and maximum (Max) values of measurement results. The normality of the result distribution was meticulously assessed using skewness and kurtosis coefficients, ensuring the validity of the findings.

The differences between educational programs on physical fitness and body composition in primary school children, while eliminating the covariates’ effect on the relationship between the groups at the initial measurement, were examined using a multivariate analysis of covariance (MANCOVA). These findings hold significant implications for the design and implementation of future educational programs. Additionally, differences between individual body composition and physical fitness variables between the control and experimental groups were assessed by analysis of covariance (ANCOVA).

## 3. Results

The research results, which relate to the effects of physical educational programs on the physical fitness and body composition in primary school children, are presented in this chapter. The obtained parameters of all analyzed results are shown in tabular format.

The normality of distribution for all variables was assessed using the Kolmogorov–Smirnov test in both the experimental and control groups. While most variables did not significantly deviate from a normal distribution (*p* > 0.05), a few variables—such as FBL, BAH, and BPT—showed significant deviations. Despite this, the use of parametric tests (e.g., independent samples *t*-tests) was considered appropriate, as each group consisted of more than 30 participants. This decision was further supported by the central limit theorem, which states that the sampling distribution of the mean tends to approximate normality with sufficiently large sample sizes, even when the data itself is not perfectly normally distributed. The application of this theorem in statistical analysis and the interpretation of results reinforces the scientific basis for using parametric tests.

**Figure 1:**
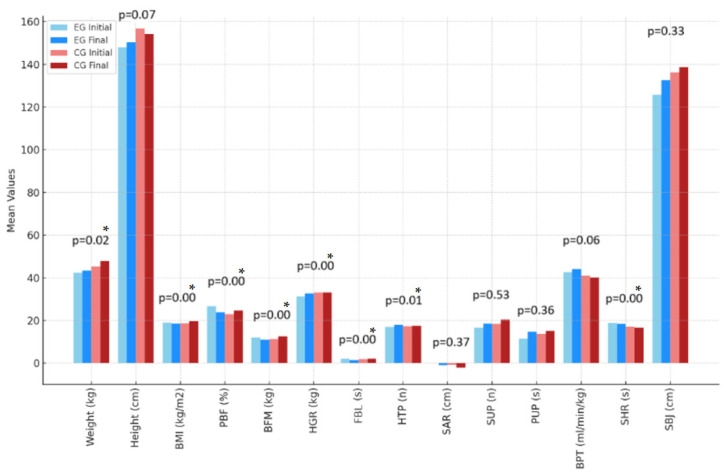
Comparison between experimental and control groups across body composition and physical fitness. Legend: BMI—body mass index; PBF—percentage of body fat; HGR—handgrip strength; FBL—flamingo balance test; HTP—hand tapping; SAR—sit-and-reach; SUP—sit-ups; PUP—pull-ups test, BPT—20 m endurance shuttle-run test; SHR—shuttle run 10 × 5 m, SBJ—standing broad jump; values marked with * indicate significant differences between initial and final measurements at *p* ≤ 0.05.

Figure 1 and Table 2 and Table 3 include information on the body composition and physical fitness variables for the experimental and control groups at the initial and final measurements. The obtained values suggested a normal distribution of the results.

The effects of the experimental program on the entire sample were applied at the multivariate level on the final measurement by a multivariate analysis of covariance (MANCOVA) while eliminating the covariates’ effect on the relationship between the groups at the initial measurement.

The results of the multivariate analysis of covariance (MANCOVA), adjusted for baseline differences, confirmed a statistically significant effect of the experimental program on the combined body composition and physical fitness variables across the entire sample (Wilks’ Lambda, *p* < 0.001, η^2^ = 0.42), indicating a large multivariate effect. In gender-specific analyses, the program demonstrated a significant multivariate impact on body composition in boys (*p* < 0.001, η^2^ = 0.38) and on both body composition (*p* = 0.01, η^2^ = 0.31) and physical fitness variables (*p* < 0.001, η^2^ = 0.35) in girls. These findings highlight the substantial and independent contribution of the acrobatics-based intervention to enhancing adolescents’ physical development, beyond initial group differences.

Further analysis of covariance (ANCOVA), which adjusted for baseline (pre-test) scores as covariates, confirmed statistically significant differences between the experimental and control groups across several body composition and physical fitness variables. In the general sample (Figure 1), significant differences were observed in weight (*p* = 0.02, η^2^ = 0.06, moderate effect), BMI (*p* = 0.00, η^2^ = 0.14, large effect), percentage of body fat—PBF (*p* = 0.00, η^2^ = 0.14, large effect), and body fat mass—BFM (*p* = 0.00, η^2^ = 0.14, large effect), all favoring the experimental group. Among boys (Table 2), similar improvements were found in BMI (*p* = 0.00, η^2^ = 0.14), PBF (*p* = 0.00, η^2^ = 0.14), and BFM (*p* = 0.00, η^2^ = 0.14). For girls (Table 3), significant improvements were noted in weight (*p* = 0.00, η^2^ = 0.14) and BFM (*p* = 0.00, η^2^ = 0.14), again in favor of the experimental group.

Regarding physical fitness outcomes, the experimental group significantly outperformed the control group in handgrip strength (HGR; *p* = 0.00, η^2^ = 0.14), balance (FBL; *p* = 0.00, η^2^ = 0.14), hand tapping (HTP; *p* = 0.01, η^2^ = 0.06), and agility as measured by the shuttle run 10×5 m (SHR; *p* = 0.00, η^2^ = 0.14) (Figure 1). These results highlight the robust effectiveness of the intervention program. Specifically, boys showed significant improvements in HGR (*p* = 0.00, η^2^ = 0.14) and SHR (*p* = 0.00, η^2^ = 0.14) (Table 2), while girls demonstrated gains in HGR (*p* = 0.00, η^2^ = 0.14), FBL (*p* = 0.00, η^2^ = 0.14), and SHR (*p* = 0.00, η^2^ = 0.14) (Table 3).

## 4. Discussion

Children who participated in the program integrating adaptive elements of sports for disabled individuals demonstrated significantly greater improvements in multiple physical fitness parameters compared to the control group. The most notable differences were observed in the flamingo balance test, handgrip, and shuttle run 10 × 5 m, favoring the experimental group. These significant results in the FBL were not only expected but also a testament to the success of the experimental group’s program, which included numerous activities involving balance, such as walking and running on a gymnastics beam, frequent falls, and recoveries with eyes open and closed.

The test results strongly depend on the motor potential of examinees, skills, and anthropometric traits [10]. For the experimental group, integrating adaptive elements of sports designed for disabled persons, such as falls, get-ups, jumps, shooting, throwing, and various gymnastic tasks, not only positively influenced their performance but also inspired a new perspective on the potential of adaptive sports programs. The experimental method, based on sports elements for disabled persons, advances children’s physical fitness, enabling them to perform complex motor activities. Teaching staff, through the implementation of the adaptive sports components, gained a renewed understanding of inclusive physical education practices. These selected exercises can effectively enhance motor development in lower primary grades [11].

The PUP testing, which includes tests for hand and shoulder strength, showed minimal improvements across both genders, suggesting weak relative strength in these muscle groups. This outcome indicates very low activation of these muscle groups in the tested children [12,13]. These findings align with studies that show that both children with disabilities and those without can exhibit similar patterns of reduced muscle strength and activation when engaging in physical activities that are not adequately adapted to their needs [14,15]. There are several specific contributing factors, including insufficient progression in the targeted exercises, the significant impact of the inherently high physical demands of the test on this age group, and the possibility that the program did not adequately address upper-body strength development, which may influence low activation. This clarification also acknowledges that while overall motor development was supported, specific strength components—particularly relative upper-body strength—require more targeted and progressive training, which was beyond the scope of the implemented program.

The weak results in the PUP test could reflect underlying issues related to muscle activation that are not solely attributable to weight gain. This underscores the need for further research to explore these variables more comprehensively and to confirm the expected outcomes, considering the broader applicability of inclusive physical activity programs [16]. Adapted physical activity programs designed for children with disabilities, which have shown benefits in body composition and strength [12,17], might also provide insights into how these programs could be advantageous for children without disabilities.

Flexibility, influenced by stretching, is not genetically conditioned and can be significantly improved [18]. Successful test results depend on various factors such as anthropometric traits, strength of body flexors, elasticity of antagonists, period of the day, temperature, and training [19]. The better flexibility of students in the control group during the experimental treatment could be influenced by uncontrolled extracurricular activities. Previous research shows that more active girls in physical education classes had better flexibility, indicating the need to emphasize activities improving this motor ability [20,21].

The experimental group’s development of cardiorespiratory endurance, measured by the BPT test, can be attributed to physiological adaptations to high aerobic demands during intensive physical activities in the experimental program. Increased anaerobic capacity was achieved mainly through sprint running at maximal speed and intensity of 70–90%, with heart rates of 160–170 beats per minute and full recovery between repetitions, incorporated into games during class [22,23].

The control group showed significantly higher values in the movement speed assessment, which uncontrolled extracurricular activities could also influence. The numerous tosses, shoots, and passes involved in adaptive sports activities might facilitate the development of these abilities. Other studies have reported similar findings on the impact of specially designed programs on children’s physical fitness [24,25].

The program’s emphasis on better organization and more advanced methodic–organizational forms during the central part of the class yielded better results in children’s physical fitness in the experimental group compared to the control group. These results align with previous studies that included various physical activities like walking, running, games, jumping, tossing, catching, shooting, crawling, and retreating [26,27].

Improvements in physical fitness were significantly lower in the control group, particularly in shuttle running (SHR). Speed and agility, assessed via shuttle running, are largely genetically conditioned (90%), limiting the potential for significant improvement within the program’s duration. Thus, the result of speed improvement could be caused unintentionally according to the duration of the experimental program [11].

Increased body mass often results from an imbalance between energy intake and consumption, indicating lifestyle habits [28]. Childhood obesity, with its health and socio-economic implications, is increasingly prevalent [29]. Significant differences in body composition were observed between the experimental and control groups, with the significant weight gain in the control group suggesting lower physical activity levels. Increased body mass in girls was correlated with increased muscle mass, likely due to the experimental treatment and improved physical fitness.

Reduced physical activity has contributed to weight gain and decreased physical fitness, increasing disease risk, consistent with prior research [30,31]. This decrease in physical activity may result from adherence to the current educational plan and program rather than the experimental one [32]. Consequently, such programs should be effective in reducing body weight, based on the positive impact of the experimental program on maintaining a normal body weight. Additionally, modifying the main phase of the class with extra exercises increases active training time, underscoring the need to enhance this phase to improve training efficiency [33]. This finding aligns with the current research.

Similarly to previous studies [7,11,15,16,17,18,25,31,34], the experimental program had a significantly higher impact on students’ physical fitness compared to the standard curriculum used in the control group [35]. The superior results of the experimental program can be attributed to the generally low level of physical activity in regular classes, the inadequate effects of standard physical education, and the limitations of the control group’s program. Furthermore, the activities used in the intervention not only support physical fitness but are also structured to enhance fundamental motor skills and overall motor competence. This framing aligns the program more closely with curricular priorities in primary education, potentially increasing its international applicability.

The study has several limitations that should be acknowledged. Some observed improvements, such as in speed and height, may be primarily influenced by genetic predispositions, reducing the extent to which they can be attributed to the intervention. This suggests that while the adaptive sports program can have a significant impact on physical fitness, there are inherent limitations to the extent of improvement that can be achieved. Additionally, flexibility and speed gains in the control group could have been affected by unmonitored extracurricular activities, introducing uncontrolled variables such as baseline differences in physical activity habits, socio-environmental factors, and individual variability in engagement with the standard PE curricula. Although extracurricular physical activities outside of school were not measured or controlled, participants were advised to maintain their usual routines and refrain from starting new physical activity programs during the study period. Nonetheless, the influence of these external factors cannot be entirely excluded. The relatively short duration of the program may have limited its impact on strength development and body composition, indicating the need for longer-term studies to fully understand the program’s effects. Furthermore, the outcomes were also heavily influenced by individual characteristics such as anthropometric traits and prior motor abilities, complicating the interpretation of the intervention’s effects. Lastly, the low activity levels and limited effectiveness of the standard physical education program used in the control group may have amplified the perceived success of the experimental intervention, highlighting the need for more effective standard programs.

This study demonstrates several notable strengths that enhance its contribution to physical education and adaptive sports programs. The innovative integration of adaptive sports elements designed for individuals with disabilities proved highly effective in improving children’s physical fitness. Significant improvements were observed in the experimental group, particularly in balance, handgrip strength, and agility, as measured by the flamingo balance test, shuttle run, and other motor tasks. The diverse range of physical activities, including falls, get-ups, jumps, and throws, fostered the development of physical fitness, while physiological adaptations indicated improved aerobic and anaerobic capacity. Positive changes in body composition, especially among girls, suggest muscle mass development associated with increased physical activity. Additionally, the improved methodological organization of the experimental sessions contributed to better outcomes, reinforcing the importance of structured and engaging class formats. Finally, the study highlights the broader applicability of inclusive programs, demonstrating that adaptive physical activities can benefit all children, regardless of physical fitness level.

## 5. Conclusions

Based on the findings of this study, the experimental program integrating adaptive elements of sports for individuals with disabilities has shown significant improvements in body composition and physical fitness parameters within the specific sample and timeframe. While these results suggest that the experimental program may offer advantages over the standard physical education classes prescribed by the Ministry of Education’s curriculum, it is important to exercise caution in generalizing these findings beyond the study context due to limitations such as sample size, study durartion, and measurement constraints. Nevertheless, this study highlights the potential of integrating adaptive physical activities into school programs, emphasizes the importance of ongoing innovation in physical education, and also highlights the program’s value in promoting both motor learning and foundational physical development at an early stage of education. Further long-term research with larger and more diverse samples are needed to validate and expand upon these findings, contributing to a more comprehensive understanding of effective interventions in this field.

## Figures and Tables

**Table 1:** Adaptive sports program.

Month	Week	Class	Learning Objective	Lesson Unit
October	I	1	Testing	Testing
	II	2	Elements of sports for individuals with disabilities: passing and throwing	Ball handling, passing the ball stationary and in motion; passing the ball with one hand and both hands
	III	3	Elements of sports for individuals with disabilities: positions and stances	Techniques for movement and positioning in defense and attack
	IV	4	Elements of sports for individuals with disabilities: situational exercises 1	Performing situational exercises, games with two goals
November	I	5	Elements of sports for individuals with disabilities: passing and throwing	Passing the ball stationary and in motion; passing the ball with one hand and both hands; passing the ball during running with one and both hands, blindfolded
	II	6	Elements of sports for individuals with disabilities: shooting techniques	Front technique shooting, rotational technique shooting
	III	7	Elements of sports for individuals with disabilities: positions and stances	Squat position, lateral lunge from kneeling, on knees
	IV	8	Elements of sports for individuals with disabilities: situational exercises 2	Performing situational exercises, games with two goals with specific tasks
December	I	9	Elements of sports for individuals with disabilities: blocking and sliding techniques	Blocking technique, sliding technique on the floor
	II	10	Elements of sports for individuals with disabilities: shooting techniques	Front technique shooting, rotational technique shooting, shooting through the legs
	III	11	Elements of sports for individuals with disabilities: penalty shooting and defense	Penalty shooting; penalty defense
	IV	12	Elements of sports for individuals with disabilities: situational exercises 3	Performing situational exercises, games with two goals, applying all techniques and rules
January	I	13	Testing	Testing
	II	14	Testing	Testing

**Table 2:** ANCOVA results for body composition and physical fitness in boys.

Variables	Experimental Group		Control Group		*p* Value	η^2^
	Initial Measurements (M ± SD)	Final Measurements (M ± SD)	Initial Measurements (M ± SD)	Final Measurements (M ± SD)		
Weight (kg)	44.58 ± 9.06	46.26 ± 10.38	47.00 ± 13.41	50.30 ± 14.34 *	0.10	0.01 (small)
Height (cm)	149.19 ± 6.88	150.47 ± 6.69 *	156.85 ± 21.00	155.30 ± 8.26	0.07	0.01 (small)
BMI (kg/m^2^)	19.59 ± 2.72	19.46 ± 3.14	19.30 ± 4.07	20.65 ± 4.40 *	0.00	0.14 (large)
PBF (%)	26.21 ± 7.22	24.10 ± 9.12 *	23.40 ± 10.19	25.45 ± 10.34 *	0.00	0.14 (large)
BFM (kg)	12.17 ± 5.33	11.74 ± 6.16	12.26 ± 8.02	13.95 ± 8.82 *	0.00	0.14 (large)
HGR (kg)	30.61 ± 5.59	32.06 ± 6.44 *	34.68 ± 7.51	34.53 ± 7.19	0.00	0.14 (large)
FBL (s)	2.17 ± 1.15	1.44 ± 0.62 *	1.70 ± 1.30	1.70 ± 1.13	0.06	0.01 (small)
HTP (n)	17.39 ± 2.23	18.72 ± 2.44*	18.35 ± 2.28	18.45 ± 1.85	0.06	0.01 (small)
SAR (cm)	2.57 ± 5.86	1.03 ± 3.74	−0.08 ± 3.76	−1.63 ± 3.3.68 *	0.18	0.01 (small)
SUP (n)	18.39 ± 4.12	20.67 ± 4.59*	21.75 ± 3.37	23.35 ± 3.22 *	0.86	0.01 (small)
PUP (s)	13.23 ± 18.59	17.07 ± 20.98 *	18.79 ± 16.81	18.92 ± 17.68	0.12	0.01 (small)
BPT (mL/min/kg)	43.75 ± 2.39	45.26 ± 2.65 *	41.00 ± 11.26	41.37 ± 11.53	0.15	0.01 (small)
SHR (s)	17.90 ± 1.63	17.67 ± 1.09	15.97 ± 1.28	15.76 ± 1.09	0.00	0.14 (large)
SBJ (cm)	132.50 ± 12.00	140.61 ± 15.02 *	146.85 ± 21.00	152.40 ± 23.12	0.66	0.01 (small)

Legend: M—Mean; SD—Standard Deviation; BMI—Body Mass Index; PBF—Percentage of Body Fat; HGR—Handgrip Strength; FBL—Flamingo Balance Test; HTP—Hand Tapping; SAR—Sit-and-Reach; SUP—Sit-Ups; PUP—Pull-Ups Test, BPT—20 m Endurance Shuttle-Run Test; SHR—Shuttle Run 10 × 5 m, SBJ—Standing Broad Jump; values marked with * indicate significant differences between initial and final measurements at *p* ≤ 0.05; η^2^—Partial Eta Squared (small effect: η^2^ = 0.01–0.05; moderate effect: η^2^ = 0.06–0.13; large effect: η^2^ ≥ 0.14).

**Table 3:** ANCOVA results for body composition and physical fitness in girls.

Variables	Experimental Group		Control Group		*p* Value	η^2^
	Initial Measurements (M ± SD)	Final Measurements (M ± SD)	Initial Measurements (M ± SD)	Final Measurements (M ± SD)		
Weight (kg)	40.65 ± 13.52	41.01 ± 12.53	43.78 ± 11.21	45.38 ± 11.29 *	0.00	0.14 (large)
Height (cm)	149.88 ± 9.38	150.55 ± 8.80	155.65 ± 24.05	154.28 ± 10.11 *	0.07	0.01 (small)
BMI (kg/m^2^)	18.56 ± 4.21	17.73 ± 3.87 *	18.10 ± 3.08	18.65 ± 3.02 *	0.11	0.01 (small)
PBF (%)	26.97 ± 8.25	23.54 ±7.50 *	22.61 ± 6.53	23.78 ± 6.77	0.71	0.01 (small)
BFM (kg)	11.95 ± 7.00	10.42 ± 6.47 *	10.38 ± 4.55	11.074.88	0.00	0.14 (large)
HGR (kg)	31.77 ± 8.52	33.09 ± 9.72 *	31.45 ± 6.00	31.55 ± 5.54	0.00	0.14 (large)
FBL (s)	2.05 ± 0.65	1.36 ± 0.66*	2.20 ± 1.51	2.30 ± 1.26	0.00	0.14 (large)
HTP (n)	16.68 ± 2.24	17.50 ± 2.54 *	16.10 ± 1.83	16.45 ± 1.70 *	0.16	0.01 (small)
SAR (cm)	−2.00 ± 4.82	−2.45 ± 3.81	−1.68 ± 7.38	−2.65 ± 7.44	0.61	0.01 (small)
SUP (n)	14.95 ± 5.49	16.86 ± 4.16 *	15.15 ± 4.69	17.45 ± 4.90	0.67	0.01 (small)
PUP (s)	9.89 ± 13.85	12.85 ± 16.95	8.67 ± 9.21	11.30 ± 12.22	0.88	0.01 (small)
BPT (mL/min/kg)	41.66 ± 2.10	43.12 ± 1.81 *	41.12 ± 1.68	39.15 ± 1.50	0.14	0.01 (small)
SHR (s)	19.56 ± 2.14	19.16 ± 2.18	18.31 ± 1.43	17.25 ± 0.79 *	0.00	0.14 (large)
SBJ (cm)	120.14 ± 20.83	126.05 ± 20.64 *	125.65 ± 24.05	124.95 ± 23.74	0.29	0.01 (small)

Legend: M—Mean; SD—Standard Deviation; BMI—Body Mass Index; PBF—Percentage of Body Fat; HGR—Handgrip Strength; FBL—Flamingo Balance Test; HTP—Hand Tapping; SAR—Sit-and-Reach; SUP—Sit-Ups; PUP—Pull-Ups Test, BPT—20 m Endurance Shuttle-Run Test; SHR—Shuttle Run 10 × 5 m, SBJ—Standing Broad Jump; values marked with * indicate significant differences between initial and final measurements at *p* ≤ 0.05; η^2^—Partial Eta Squared (small effect: η^2^ = 0.01–0.05; moderate effect: η^2^ = 0.06–0.13; large effect: η^2^ ≥ 0.14).

## Data Availability

The datasets used and/or analyzed during the current study are available from the corresponding author on reasonable request.

## Abbreviations

The following abbreviations are used in this manuscript:

BW	Body weight
BH	Body height
BMI	Body mass index
PBF	Percentage of body fat
HGR	Handgrip strength
FBL	Flamingo balance test
HTP	Hand tapping
SAR	Sit-and-reach
SUP	Sit-ups
PUP	Pull-ups test
BPT	Beep test/The 20 m endurance shuttle run test
SHR	Shuttle run 10 × 5 m
SBJ	Standing broad jump
